# Supplementary material for: A generic construction for revocable identity-based encryption with subset difference methods
Source: PLoS One. 2020 Sep 18;15(9):e0239053. doi: 10.1371/journal.pone.0239053 (PMC7500651; doi:10.1371/journal.pone.0239053)
Supplement: Attachment — Submitted filename: reply-letter.pdf [file pone.0239053.s001.pdf]

Authors' Reply

We appreciate the detailed comments of the reviewers. This is our reply letter for the comments of the reviewers.

Reviewer #1's Comments

Q1) In this work, the authors design a Revocable Identity-Based Encryption RIBE scheme by combining IBE, identity-based revocation (IBR), and two-level HIBE schemes. Overall, the work has technical depth and has potential. The solution is described in a pure mathematical and technical way, which makes it hard to grasp it for non-expert readers. I suggest adding a paragraph to explain the solution in plaintext in an accessible manner.

A1) Thank you for the comments. In Introduction, we explained the design principle of our generic RIBE scheme.

Q2) It would be quite interesting to compare the proposed solution with regards to other security solutions such as trust. In this context, I suggest comparing with the following articles: Towards trustworthy multi-cloud services communities: A trust-based hedonic coalitional game. IEEE Transactions on Services Computing, 11(1), 184-201, 2016. A survey on trust and reputation models for Web services: Single, composite, and communities. Decision Support Systems, 74, 121-134, 2015. Trust management for secure cognitive radio vehicular ad hoc networks. Ad Hoc Networks, 86, 154-165, 2019. A lightweight trust management algorithm based on subjective logic for interconnected cloud computing environments. The Journal of Supercomputing, 75(7), 3534-3554, 2019.

A1) Thank you for the suggestion of related work. Unfortunately the solutions of these articles are not directly compared with our solution since our solution is a cryptographic system that preserves the privacy of messages with additional properties.

Q3) The authors should present some experimental results to validate the performance of the solution.

A1) Thank you for the comments. In Table 1, we compared our solution with previous RIBE solutions. We didn't present the experimental results since we do not claim that our solution is more efficient than directly constructed RIBE schemes. Instead, we claim that a generic RIBE scheme by using the SD method can be built by combining HIBE and IBR schemes.

Reviewer #2's Comments

Q1) Based on my own reading of manuscript no.PONE-D-20-11744, I think that there is not enough novelty in the paper, most of the contribution has been published in the following paper: [1].

A1) The reviewer mentioned that our manuscript is already published in another journal. However, the mentioned paper [1] is our paper and it is stored in the IACR ePrint Achieve, which is a preprint server, for sharing our work. The criteria for publication of PLOS ONE also mentioned that the publication on a preprint server does not impact consideration of the manuscript at any PLOS journal.

References

[1] Kwangsu Lee: A Generic Construction for Revocable Identity-Based Encryption with Subset Difference Methods. IACR Cryptol. ePrint Arch. 2019: 798 (2019)
