# Supplementary material for: A generic construction for revocable identity-based encryption with subset difference methods
Source: PLoS One. 2020 Sep 18;15(9):e0239053. doi: 10.1371/journal.pone.0239053 (PMC7500651; doi:10.1371/journal.pone.0239053)
Supplement: Attachment — Submitted filename: reply-letter.pdf [file pone.0239053.s003.pdf]

Authors' Reply

We appreciate the detailed comments of the reviewers. This is our reply letter for the comments of the reviewers.

Editor's Comments

Q1) The paper is based on the work published by Inscrypt 2019 which was also a good research work. I can see the contributions of the paper but the motivations of the paper are not explicitly described. One paragraph of motivations is necessary to show the importance of the research work and also attract more readers in future.

A1) Thanks for your point. We explicitly stated the motivation of our study in the introduction section.

Reviewer #3's Comments

Q1) I suggest that the section 1.2 should be demonstrated before the concrete construction.

A1) We took what the reviewer pointed out and moved the our technique section before the generic construction section.

Q2) Compared with the reference 15, what are the advantages of proposed generic RIBE scheme?

A2) Our generic RIBE scheme and the LLP-RIBE scheme [1] are the same in terms of using the SD method. However, since our generic construction uses IBE, IBR, and HIBE schemes as a black box to design an RIBE scheme, it is possible to design an RIBE scheme by using any IBE, IBR, and HIBE scheme (in bilinear maps or lattices). In contrast, the LLP-RIBE scheme can be designed only using the IBE scheme which has a specific mathematical structure in the bilinear map since it is not a black box construction.

Reviewer #4's Comments

Q1) In the Definition 2.5 (Hierarchical Identity-Based Encryption, HIBE), what's the relation between $ID|_\ell$, $ID|_k$ and $ID'|_k$? Dose the $ID|_\ell$ denote a subset of $ID|_k$ or the opposite? Is the $ID'|_k$ the last identity of hierarchical identity $ID|_k$ or some identity of $ID|_k$?

A1) The definition of HIBE in the previous manuscript was insufficient, so we revised the definition of HIBE to reflect what the reviewers pointed out in the revised manuscript. We explain it using the modified notation. Here, a hierarchical identity $HID|_k$ is expressed as an identity vector in the form of $(ID_1, \ldots, ID_k)$ where k represents the size of this vector. The hierarchical identity $HID|_k = (ID_1, \ldots, ID_k)$ must be a prefix of the hierarchical identity $HID|_\ell = (ID_1, \ldots, ID_\ell)$ to satisfy the correctness requirement of the HIBE scheme.

Q2) In RIBE.Setup (Section 3.3), "It defines a binary tree BT by running SD.Setup(2^n) where an identity $ID \in I$ is uniquely assigned to a leaf node v such that $Label(v) = ID$." What does the identity "ID" point to? Do the authors mean all the identities that users use are pre-set by the key generation center?

A2) For example, suppose that $n = 3$. In this case, the binary tree has 8 leaf nodes, and each leaf node has a label of $000, 001, 010, \ldots, 111$ using the *Label* function. At this time, since an identity ID is also expressed as a 3-bit binary number, the identity $ID = 000$ is assigned to the leftmost node in the binary tree, and the identity $ID = 111$ is assigned to the rightmost node in the binary tree. Therefore, if an identity ID is given, the location of the leaf node in the binary tree is uniquely determined. Note that the key generation center does not pre-set the location of each identity.

Q3) The authors mention that "the size of an update key is reduced but the size of a ciphertext is increased.". It is an interesting improvement. I suggest the authors could provide concrete analysis on computation and storage complexity or even some experimental implementation in the Section 5. This will make readers fully understand this paper's innovation.

A3) We appreciate the reviewer's point. In Section 5, we analyzed the actual size of the private key, update key, and ciphertext of our generic RIBE scheme when our RIBE scheme with the LSD method is instantiated by using IBE, IBR, and HIBE schemes in the MNT asymmetric bilinear group.

References

[1] Lee K, Lee DH, Park JH. Efficient revocable identity-based encryption via subset difference methods. Des Codes Cryptogr. 2017;85: 39-76.
