# Supplementary material for: A generic construction for revocable identity-based encryption with subset difference methods
Source: PLoS One. 2020 Sep 18;15(9):e0239053. doi: 10.1371/journal.pone.0239053 (PMC7500651; doi:10.1371/journal.pone.0239053)
Supplement: Attachment — Submitted filename: PONE-D-20-11744_R1_review.docx [file pone.0239053.s002.docx]

**Paper strengths.**

This manuscript mainly focuses on the demand of key revocation for identity-based encryption, and proposes a revocable solution with the subset difference method. Their IBE scheme combine identity-based revocation (IBR), and two-level HIBE schemes as basic building blocks.

Strength:

1) The motivation and idea are good, and the proposed RIBE approach seems to be innovative and practical.

2) The security definition and formal proof of designed RIBE is very nice.

3) The manuscript is presented in an intelligible fashion and is written in standard English.

**Paper weaknesses.**

There is no implementation or complexity analysis to indicate the feasibility of the scheme.

**Detailed review.**

1). In the Definition 2.5 (Hierarchical Identity-Based Encryption, HIBE), what's the relation between ID|\ell, ID|k and ID'_k? Dose the ID|\ell denote a subset of ID|k or the opposite? Is the ID'_k the last identity of hierarchical identity ID|k or some identity of ID|k ?

2). In RIBE.Setup (Section 3.3), “It defines a binary tree BT by running SD.Setup(2n) where an identity ID \in I is uniquely assigned to a leaf node v such that Label(v) = ID.” What does the identity “ID” point to? Do the authors mean all the identities that users use are pre-set by the key generation center?

3). The authors mention that “the size of an update key is reduced but the size of a ciphertext is increased.”. It is an interesting improvement. I suggest the authors could provide concrete analysis on computation and storage complexity or even some experimental implementation in the Section 5. This will make readers fully understand this paper's innovation.
